# Acute and subchronic toxicity study of nonpolar extract of licorice roots in mice

**DOI:** 10.1002/fsn3.1465

**Published:** 2020-04-08

**Authors:** Hyun‐Yong Kim, Guanglei Zuo, Soo Kyeong Lee, Soon Sung Lim

**Affiliations:** ^1^ Department of Food Science and Nutrition Hallym University Chuncheon Korea; ^2^ Institute of Korean Nutrition Hallym University Chuncheon Korea

**Keywords:** acute toxicity, de‐glycyrrhizin, licorice, nonpolar extract, subchronic toxicity, toxicology

## Abstract

Licorice is used as a medicinal plant, and several studies have shown that licorice has beneficial effects. The objective of this study was to evaluate the safety of nonpolar licorice extract using toxicity experiments. Nonpolar extract from the root of *Glycyrrhiza uralensis* (NERG) was analyzed by high‐performance liquid chromatography (HPLC). Antioxidant ability was determined by method of TPC and DPPH. Blood pressure was monitored by using blood pressure meter. In the acute study, a single dose (2,000 mg/kg) was orally administered to mice. In the subchronic study, mice were treated with extract at doses (50, 100, 500, and 1,000 mg/kg) for 120 days. Significantly difference was not shown at blood pressure, hematological, and biochemical parameters, and histopathology on mice. The results suggested that at acute and subchronic toxicity, each levels of nonpolar licorice extract administration in experiments did not cause toxicity effects or death on mice.

## Graphical abstract

In the acute study, a single dose (2,000 mg/kg) was orally administered to mice. In the subchronic study, mice were treated with extract at doses (50, 100, 500, and 1,000 mg/kg) for 120 days. The results suggested that at acute and subchronic toxicity, each levels of nonpolar licorice extract administration in experiments did not cause toxicity effects or death on mice.

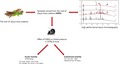

## 1 INTRODUCTION

Licorice originates from the dried root of *Glycyrrhiza uralensis*, and it has medicinal value against diseases, such as rheumatic and other types of pain (Haraguchi, Ishikawa, Mizutani, Tamura, & Kinoshita, 1998). Many studies have found pharmacologically important phenolic compounds in licorice (Yoon, Jung, & Cheon, 2005), and active ingredients of licorice like saponin and flavonoids have been shown to have various therapeutic activities (Sun, Xie, & Liu, 2007). The reported major compounds of licorice are saponins like glycyrrhizin and glycyrrhetinic acid, and pharmacological properties of each compound demonstrated anti‐inflammatory, radical scavenging activity, and protective effect on ischemia/reperfusion or nephrotoxic injury, respectively (Hwang et al., 2006). Glycyrrhizin is the major saponin compound isolated from licorice root (Yoshida et al., 2007), and it has been proven to be effective in protecting against liver disease (Wang, Guo, Li, Liu, & Zern, 1998). However, glycyrrhizin has also been reported to induce hypertension (Isbrucker & Burdock, 2006). For this reason, we extracted licorice using a nonpolar solvent system in order to reduce the content of glycyrrhizin. Deglycyrrhizinated licorice (DGL) was developed to provide the therapeutic benefits of licorice to patients without its harmful side effects (Dastagir & Rizvi, 2016). Additionally, DGL showed effect of alleviation of ulcer in gastric mucosal damage in rats (Raveendra et al., 2012) and chewable DGL licorice tablets are used for health supplement (Thakur & Raj, 2017). Adverse effects of medicinal plants have been reported, however, raising concerns about the potential toxic effects from chronic use (Porwal, Khan, & Maheshwari, 2017). Since information about the toxicity of licorice extract is limited, it is important to conduct studies to assess the safety of nonpolar licorice root extract. Therefore, the objective of this study was to evaluate the safety of nonpolar licorice root extract in mice by utilizing acute and subchronic toxicity tests. Our aim is to provide valuable information regarding the usage of this extract.

## 2 MATERIALS AND METHODS

### 2.1 Plant material, preparation of extraction, and administration of extract

The root of licorice was obtained from Deagwang korean plant sales, Chuncheon, Korea. The specimen of *G. uralensis* was confirmed to Seoul national university and deposited in the Regional Innovation Center (RIC; #040322) of the Hallym University, Chuncheon. The plant root was air‐dried and then soaked in a mixture of solvent (Hexane:Ethanol = 9:1) for 24 hr. After 24 hr, the extraction was filtered and concentrated at 37°C using a rotary evaporator (Vacuubrand, CVC 3000). The nonpolar extract from the root of *G. uralensis* (NERG) was administered orally, in the shape of a pellet, for the acute and subchronic toxicity studies.

### 2.2 Chemical and reagents

All chemicals were analytical grade. Acetonitrile was acquired from J. T. Baker Chemical Company. Acetic acid, Folin–Ciocalteu phenol reagent, and 2,2‐diphenyl‐1‐picrylhydrazyl (DPPH) were purchased from Sigma Chemical Company.

### 2.3 Phytochemical characterization: high‐performance liquid chromatography (HPLC)

Chromatographic analysis of nonpolar compounds was performed using an HPLC system (Dionex). The separations were carried out under gradient conditions with a Phenomenex Luna C18 column (4.6 μm × 25 cm, with 5 μm particle size) at 28°C. The mobile phase was water containing 2% acetic acid, and acetonitrile (B), according to the following elution program: 20% B (0–5 min); 20%–90% B (5–55 min); 90%–100% B (55–60 min); 100%–20% B (60–62 min); 20% B (62–65 min). The flow rate was 0.8 ml/min, and the injection volume was 10 μl. Two hundred fifty‐four nanometer wavelength was used for UV detection.

### 2.4 Total phenolic content and DPPH

The determination of total phenolic content of the nonpolar extract from the root of *Glycyrrhiza uralensi* (NERG) was performed by the Folin–Ciocalteu method. The samples were read at 765 nm in a spectrophotometer. The total phenolic content was expressed in milligram of gallic acid equivalent (GAE) per gram of plant extract. The equation obtained from the standard curve of gallic acid (0.016–1 mg/ml) was *y* = 1.3832*x* + 0.0242 (*R* ^2^ = .999). The experiment was conducted in triplicate.

The radical scavenging activity of NERG was quantified using DPPH, as described previously, with a slight modification (Quispe, Hwang, Wang, Zuo, & Lim, 2017). One hundred eighty microliter of DPPH solution (0.32 mM in methanol) was mixed with 30 μl of sample. After a 15‐min incubation in the dark room, the change in absorbance was measured at 570 nm on a microplate reader (EL800 Universal Microplate reader, BioTek Instruments). DPPH radical scavenging activity was calculated as % inhibition using the formula: % DPPH inhibition = [1 − (As − Ab/Ac − Ab)] × 100% (Ac is the absorbance of the DPPH solution [180 μl] with methanol [30 μl]; Ab is the absorbance of distilled water [180 μl] with methanol [30 μl]; and As is the absorbance of DPPH solution [180 μl] with sample solution [30 μl]).

### 2.5 Animals

The male ICR mice (30 g ± 3 g; 5 weeks old) were purchased from Central Lab Animal (SLC Inc.). Mice were separated into four groups by dose treatment (50, 100, 500, and 1,000 mg/kg of body weight) under standard laboratory conditions with a 12 hr light/dark cycle. The animal room temperature was maintained at 25°C ± 2°C, with a relative humidity of 55% ± 5%. Food and water were provided ad libitum. All experimental procedures were approved by the Committee on Animal Experimentation of Hallym University and performed in compliance with the University's Guidelines for the Care and Use of Laboratory Animals (Hallym2017‐55). The mice were cared for in accordance with the institutional ethical guidelines.

### 2.6 Blood pressure monitoring

The blood pressure was measured by determining the systolic blood pressure at the artery in the tail of the mice with an indirect blood pressure meter (LE 5002, Panlab) for 7 weeks, before measuring toxicity. An average blood pressure was taken from three separate measurements (NERG dosing were 50 mg, 100 mg/kg). In order to reduce the error of the measurement, the mice were allowed to adapt to the plastic device of the measuring machine for 30 min each day over the course of 1 week before blood pressure measurements were started.

### 2.7 Acute toxicity

According to OECD 423 (The Organization for Economic Cooperation and Development [OECD] Test Number 423: Acute Oral Toxicity—Acute Toxic Class Method guidelines), mice were orally treated with a single dose of NERG (2,000 mg/kg of body weight dissolved in distilled water; *n* = 10). The control group was treated with the vehicle alone (distilled water). After the single‐dose administration, body weight, signs of toxicity, and mortality were observed. All mice were sacrificed for necropsy examination, and organs were weighed.

### 2.8 Subchronic toxicity

Animals were divided into four groups (*n* = 10 per group), and body weight was recorded during the treatment period (120 days) based on the method of subchronic 4‐month oral toxicity study (Genta, Cabrera, Grau, & Sanchez, 2005). Groups were treated with NERG at doses of 50 mg/kg of body weight (low dose), 100 mg/kg of body weight, 500 mg/kg of body weight (both as middle doses), and 1,000 mg/kg of body weight (high dose) by mixing NERG with mouse chow. The control group was fed normal mouse chow with no NERG. Mice were observed for abnormalities during the treatment period and were also anesthetized for blood collection for hematologic and biochemical analysis. At the conclusion of the experiment, liver, kidney, spleen, lung, and testis were removed and weighed in order to calculate the relative weight. The organs were then fixed in 10% formalin and processed for histological analysis.

### 2.9 Hematological and biochemical analysis

Blood was centrifuged (1,200 *g* for 30 min, 4°C) to obtain serum. Serum was analyzed for the levels of albumin, alkaline phosphate, alanine aminotransferase (ALT), aspartate aminotransferase (AST), blood urea nitrogen (BUN), calcium, cholesterol, HDL‐cholesterol, creatinine, direct bilirubin, glucose, iron, magnesium, total bilirubin, triglycerides, and total protein. Total blood was analyzed for hematological parameters, including white blood cells (WBC), neutrophils (NE), lymphocytes (LY), monocytes (MO), eosinophils (EO), basophils (BA), red blood cells (RBC), hemoglobin (Hb), hematocrit (HCt), mean corpuscular volume (MCV), mean corpuscular hemoglobin (MCH), and mean corpuscular hemoglobin concentration (MCHC).

### 2.10 Histopathological analysis

Organs (heart, liver, kidney, spleen, lung, and testis) were fixed in 10% formalin, paraffin‐embedded, sectioned at 5 μm thickness, deparaffinized, rehydrated, and stained with hematoxylin and eosin (H&E). Histological analysis was performed by light microscopy examination (OM, Axio Imager A1, Carl Zeiss), the magnification power used was 100× and 200×.

### 2.11 Statistical analysis

All values are expressed as the mean ± *SD*. Comparison between groups was performed using Student's *t* test. A *p* ≤ .05 was considered statistically significant.

## 3 RESULTS

### 3.1 HPLC analysis

**Figure 1:**
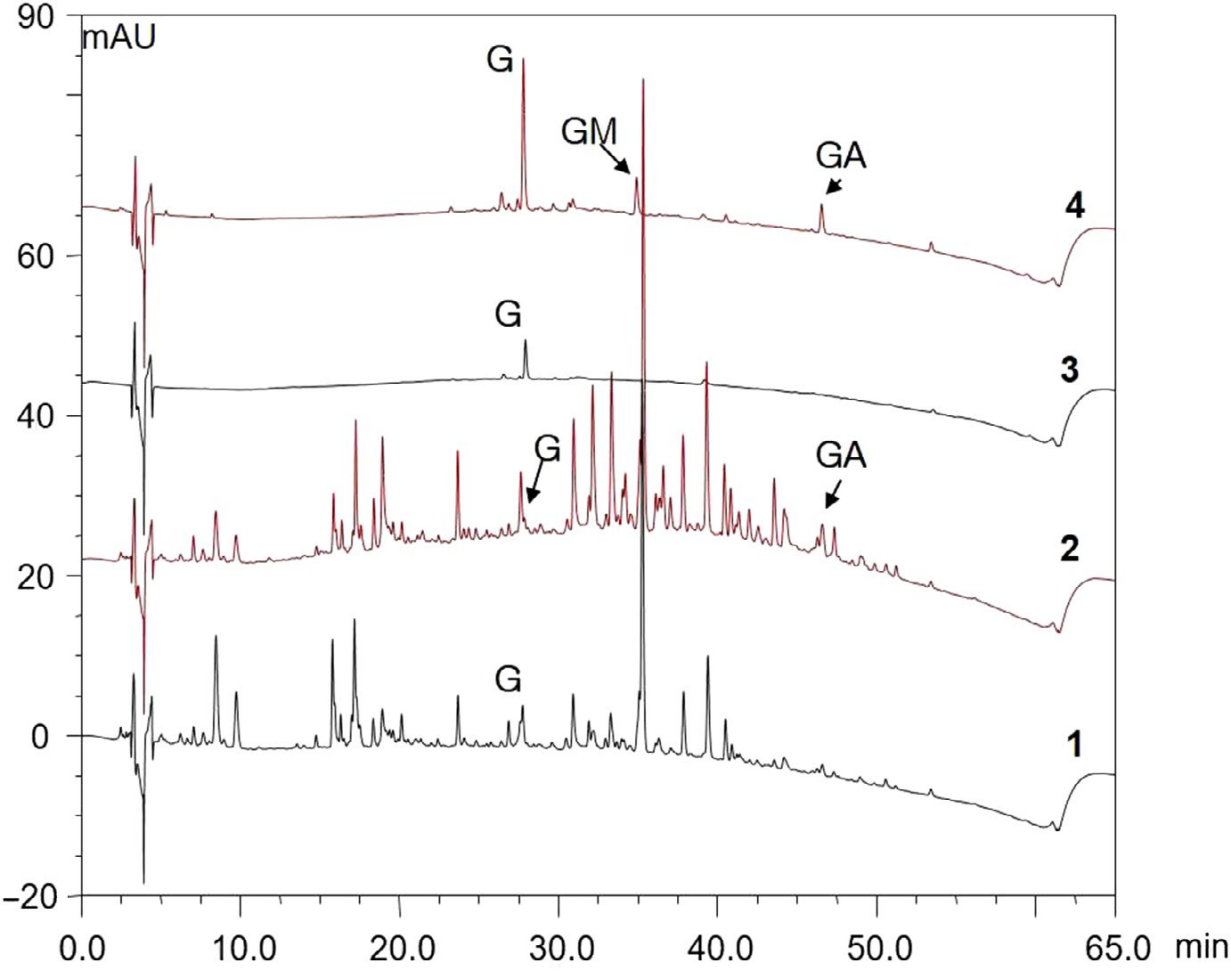
HPLC chromatogram of NERG

In our previous study, NERG was analyzed by HPLC (Hwang et al., 2006). Figure 1 shows nonpolar compounds in the HPLC purification of licorice extract, which was used in this study. Glycyrrhizin, glycyrrhetinic acid monoglucuronide, and glycyrrhetinic acid were the major compounds identified in NERG.

### 3.2 Total polyphenolic content and DPPH

Nonpolar extract from the root of *G. uralensis* showed a concentration of total polyphenols of 0.09 ± 0.4 mg/g GAE. Antioxidant activity as determined by DPPH was 73.23% ± 0.2 inhibition % (data not shown).

### 3.3 Blood pressure

The systolic blood pressure, diastolic blood pressure, and heart rate in ICR mice treated with NERG (500 and 1,000 mg) were not significantly different than those in the control‐treated mouse group (Table 1). These results suggest that the reduction of glycyrrhizin contents through nonpolar extraction of licorice inhibited influence at blood pressure.

**Table 1:** Blood pressure of ICR mice orally administered with NERG

NERG concentration	Systolic blood pressure(mmHg)	Diastolic blood pressure (mmHg)	Heart rate(PBM)
Control	129.36 ± 6.34	98.05 ± 6.99	636.61 ± 42.25
500 mg	130.40 ± 5.46	105.78 ± 6.01	653.24 ± 46.78
1,000 mg	119.72 ± 4.91	92.47 ± 5.91	676.77 ± 54.49

### 3.4 Acute toxicity

The acute toxicity test, which was performed by oral administration of NERG at a dose of 2,000 mg/kg, did not show any mortality or toxicity during the treatment period (data not shown). The body weights of control and NERG‐treated groups were similar (data not shown). No changes were found in the weights of the organs (data not shown).

### 3.5 Subchronic toxicity

**Figure 2:**
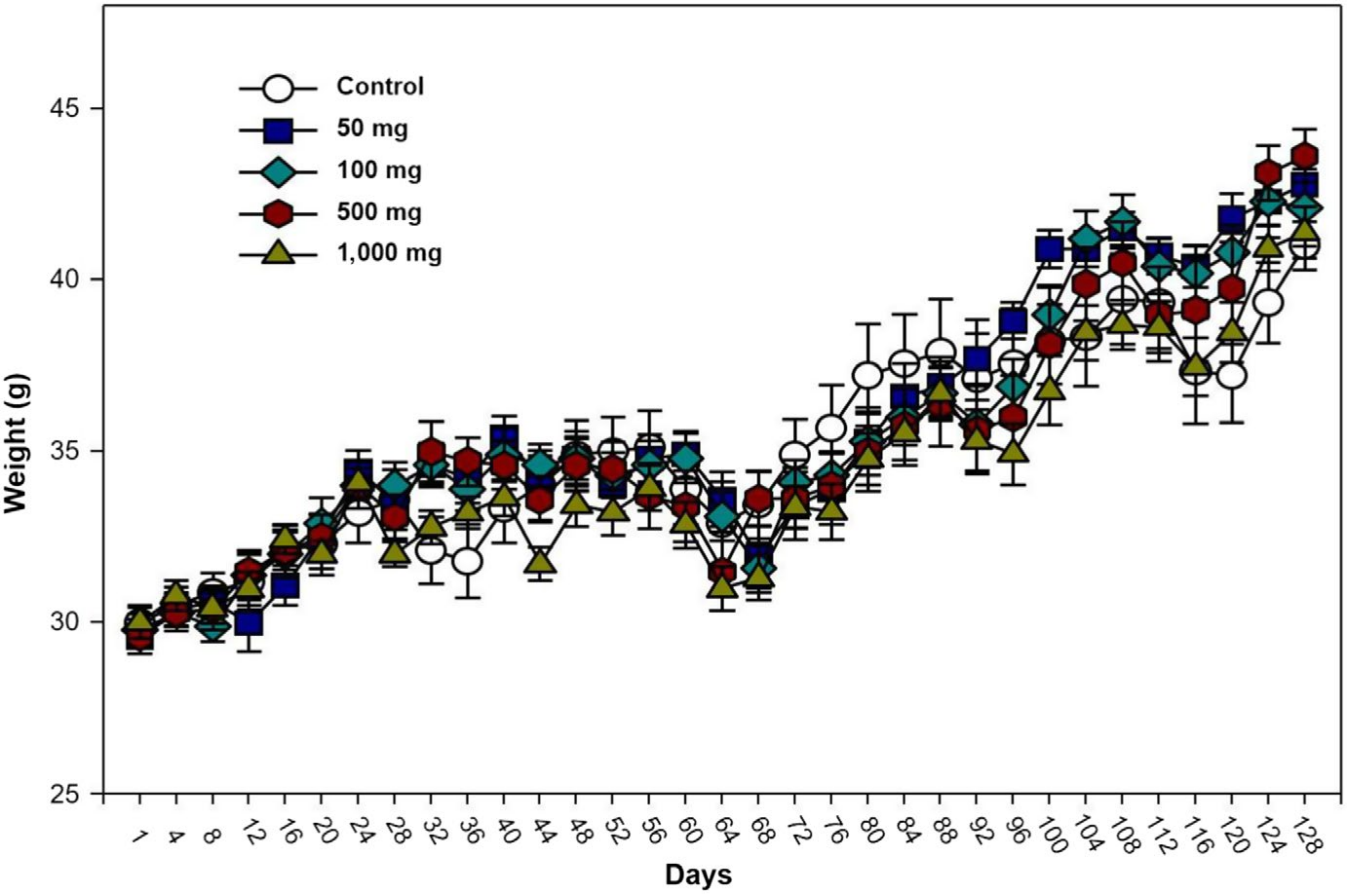
Body weight changes of ICR mice orally administered with NERG for 120 days

During 120 days of NERG oral treatment (50, 100, 500, and 1,000 mg/kg), there were no abnormal changes in body weight (Figure 2). Furthermore, there were no signs of adverse effects (data not shown) and no death occurred during the 120 days of treatment (data not shown). While the NERG‐treated group showed increased liver weight when compared to the control group, there was no significant difference in the weight of any other organ in the NERG‐treated group as compared to the control group (Table 2). Serum biochemistry is summarized in Table 3. Considering the biochemical parameters evaluated, we observed the reduction of alkaline phosphatase and ALT activity in the serum of the NERG‐treated group when compared to the control group. There were no significant changes in the levels of AST, BUN, creatinine, direct bilirubin, glucose, magnesium, total bilirubin, triglycerides, or total protein. NERG treatment (1,000 mg/ml) increased the serum levels of albumin, calcium, cholesterol, HDL‐cholesterol, and iron levels (Table 3). The effect of NERG on hematological parameters is shown in Table 4. The NERG‐treated group had significantly decreased LY and MO values. However, there were no significant changes in WBC, NE, EO, BA, RBC, HB, HCt, MCV, MCH, or MCHC values between the control and NERG‐treated group (Table 4).

**Table 2:** Organ weight of ICR mice orally administered with NERG for 120 days

Parameter	Control	50 mg	100 mg	500 mg	1,000 mg
Liver	1.77 ± 0.09	2.39 ± 0.11	2.02 ± 0.07	2.28 ± 0.05	2.34 ± 0.08
Lung	0.28 ± 0.01	0.27 ± 0.01	0.26 ± 0.01	0.28 ± 0.01	0.28 ± 0.01
Heart	0.21 ± 0.01	0.23 ± 0.01	0.22 ± 0.006	0.23 ± 0.00*	0.23 ± 0.01
Kidney	0.33 ± 0.02	0.35 ± 0.01	0.34 ± 0.01	0.34 ± 0.01	0.34 ± 0.01
Spleen	0.14 ± 0.01	0.12 ± 0.01	0.12 ± 0.02	0.14 ± 0.01	0.15 ± 0.01
Testis	0.14 ± 0.01	0.14 ± 0.01	0.14 ± 0.01	0.13 ± 0.01	0.14 ± 0.01

* The values were considered to be significantly different when *p* < .05.

**Table 3:** Biochemical serum values of ICR mice orally administered with NERG for 120 days

Parameter	Unit	Control	50 mg	100 mg	500 mg	1,000 mg
Albumin	g/dl	1.77 ± 0.1	2.39 ± 0.11	2.02 ± 0.07	2.33 ± 0.10*	2.27 ± 0.06*
Alkaline phosphatase	mg/dl	108.0 ± 14.0	79.25 ± 4.76	73.89 ± 5.37*	77.50 ± 5.42*	79.40 ± 5.12*
ALT	mg/dl	69.33 ± 15.3	48.89 ± 7.44	37.78 ± 4.23*	29.33 ± 5.05*	61.0 ± 16.11
AST	mg/dl	117.33 ± 12.9	92.52 ± 11.25	102.0 ± 18.46	95.17 ± 18.97	126.0 ± 13.35
BUN	mg/dl	19.37 ± 0.10	22.55 ± 0.76	20.38 ± 1.42	21.32 ± 1.22	18.34 ± 0.79
Calcium	mg/dl	5.87 ± 0.4	7.55 ± 0.13	6.23 ± 0.26	6.87 ± 0.24	7.31 ± 0.09*
Cholesterol	mg/dl	73.03 ± 2.7	99.33 ± 5.96	85.87 ± 7.99	96.42 ± 8.02	102.76 ± 4.9*
Creatinine	mg/dl	0.30 ± 0.06	0.29 ± 0.01	0.32 ± 0.01	0.32 ± 0.03	0.31 ± 0.01
Direct bilirubin	mg/dl	0.27 ± 0.03	0.29 ± 0.03	0.45 ± 0.19	0.17 ± 0.02*	0.23 ± 0.03
Glucose	mg/dl	179.33 ± 16.4	153.53 ± 8.4	218.44 ± 13.4	175.33 ± 33.1	277.0 ± 24.6
HDL‐Chol	mg/dl	55.00 ± 1.00	91.32 ± 5.66	72.67 ± 7.19	82.50 ± 6.04*	91.2 ± 4.2*
Iron	mg/dl	134.00 ± 60.5	247.25 ± 8.4	203.78 ± 15.2	240.17 ± 25.1	229.70 ± 12.7*
Magnesium	mg/dl	1.90 ± 0.1	2.03 ± 0.05	2.18 ± 0.11	2.13 ± 0.11	2.32 ± 0.15
Total bilirubin	mg/dl	0.20 ± 0.02	0.24 ± 0.02	0.34 ± 0.09	0.18 ± 0.02	0.25 ± 0.04
Triglyceride	mg/dl	76.33 ± 11.7	118.94 ± 10.3	56.00 ± 5.78	90.0 ± 9.33	109.33 ± 12. 7
Total protein	g/dl	2.70 ± 0.1	3.36 ± 0.2	2.97 ± 0.17	3.37 ± 0.07*	3.74 ± 0.10*

* The values were considered to be significantly different when *p* < .05.

**Table 4:** Hematological findings of ICR mice orally administered with NERG for 120 days

Parameter	Unit	Control	50 mg	100 mg	500 mg	1,000 mg
Leukocyte						
WBC	K/μl	8.06 ± 0.87	5.75 ± 0.73	4.62 ± 0.55*	4.01 ± 0.79*	5.01 ± 0.88
NE	K/μl	1.87 ± 0.33	2.58 ± 0.49	1.51 ± 0.37	1.80 ± 0.38	2.33 ± 0.51
LY	K/μl	5.52 ± 0.39	2.91 ± 0.49*	2.86 ± 0.37*	2.00 ± 0.34*	2.43 ± 0.38*
MO	K/μl	0.55 ± 0.13	0.21 ± 0.04*	0.11 ± 0.02*	0.10 ± 0.03*	0.20 ± 0.06*
EO	K/μl	0.09 ± 0.05	0.04 ± 0.008	0.02 ± 0.007*	0.08 ± 0.05	0.04 ± 0.008
BA	K/μl	0.03 ± 0.01	0.01 ± 0.004	0.01 ± 0.002*	0.02 ± 0.02	0.01 ± 0.004
Erythrocyte						
RBC	M/μl	8.67 ± 0.19	8.44 ± 0.27	7.87 ± 0.14*	7.66 ± 0.18*	8.21 ± 0.17
Hb	g/μl	13.40 ± 0.17	12.98 ± 0.39	11.80 ± 0.21*	11.66 ± 0.25*	12.48 ± 0.34
HCT	%	45.00 ± 1.81	43.12 ± 1.31	38.84 ± 0.58*	38.41 ± 0.099*	41.32 ± 1.17
MCV	fl	51.90 ± 1.85	51.15 ± 0.67	49.47 ± 1.04	50.20 ± 0.68	50.34 ± 0.85
MCH	Pg	15.47 ± 0.24	15.39 ± 0.17	15.02 ± 0.26	15.24 ± 0.15	15.20 ± 0.24
MCHC	g/dl	29.83 ± 0.82	30.13 ± 0.36	30.40 ± 0.33	30.36 ± 0.41	30.20 ± 0.21

Abbreviations: BA, basophil; EO, eosinophil; HB, hemoglobin; HCT, hematocrit; LY, lymphocyte; MCH, mean corpuscular hemoglobin; MCHC, mean corpuscular hemoglobin concentration; MCV, mean corpuscular volume; MO, monocyte; NE, neutrophil; RBC, red blood cell; WBC, white blood cell.

* The values were considered to be significantly different when *p* < .05.

### 3.6 Histopathology

**Figure 3:**
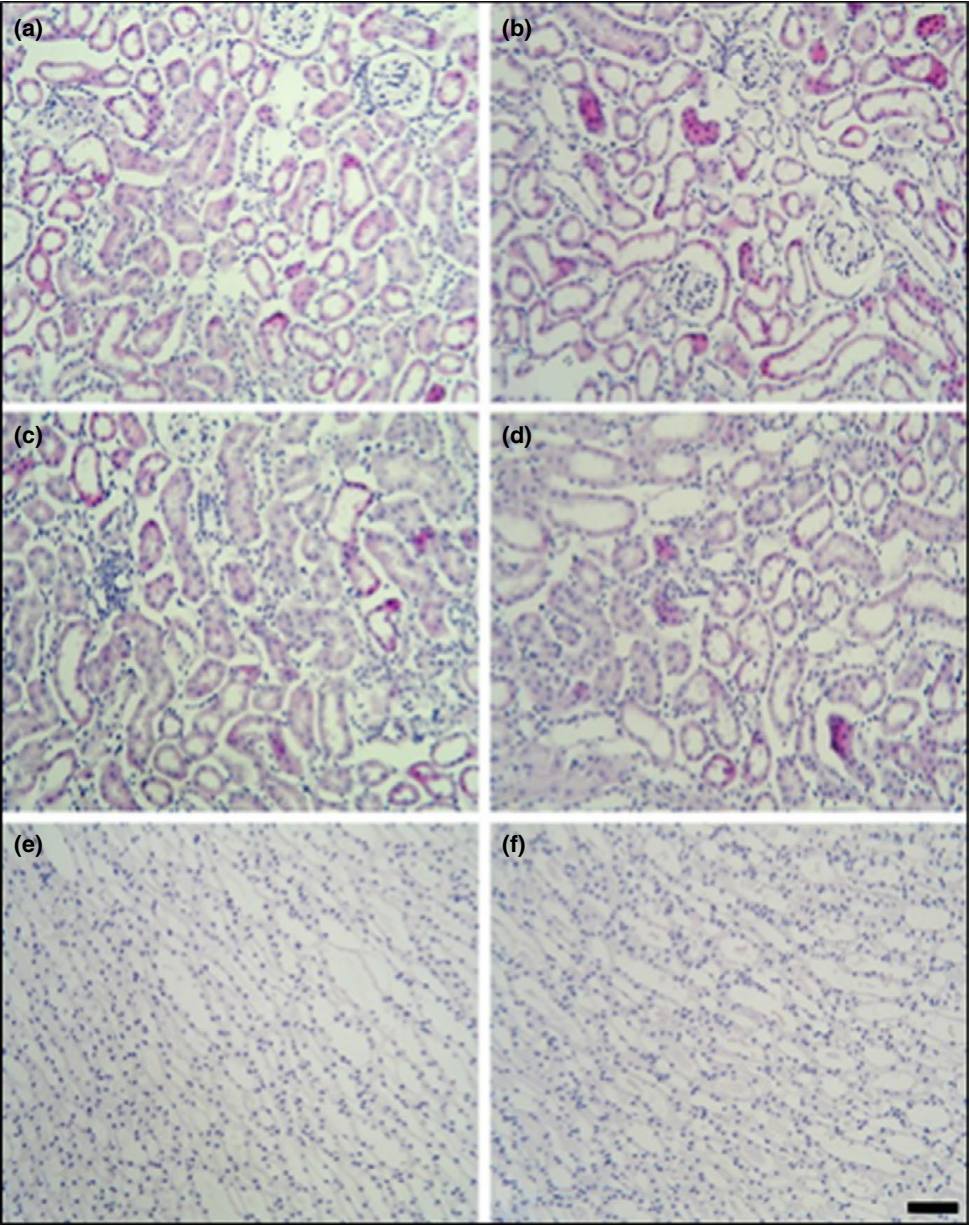
Photomicrography of kidneys of the mice of the control group (a, c, e) and treated with 1,000 mg/kg groups (b, d, f). Light micrographs of cortex (a, b), outer medulla (c, d) and inner medulla (e, f) of kidney. Magnification: a–e: ×200. Scale bars: a–e: 50 μm

**Figure 4:**
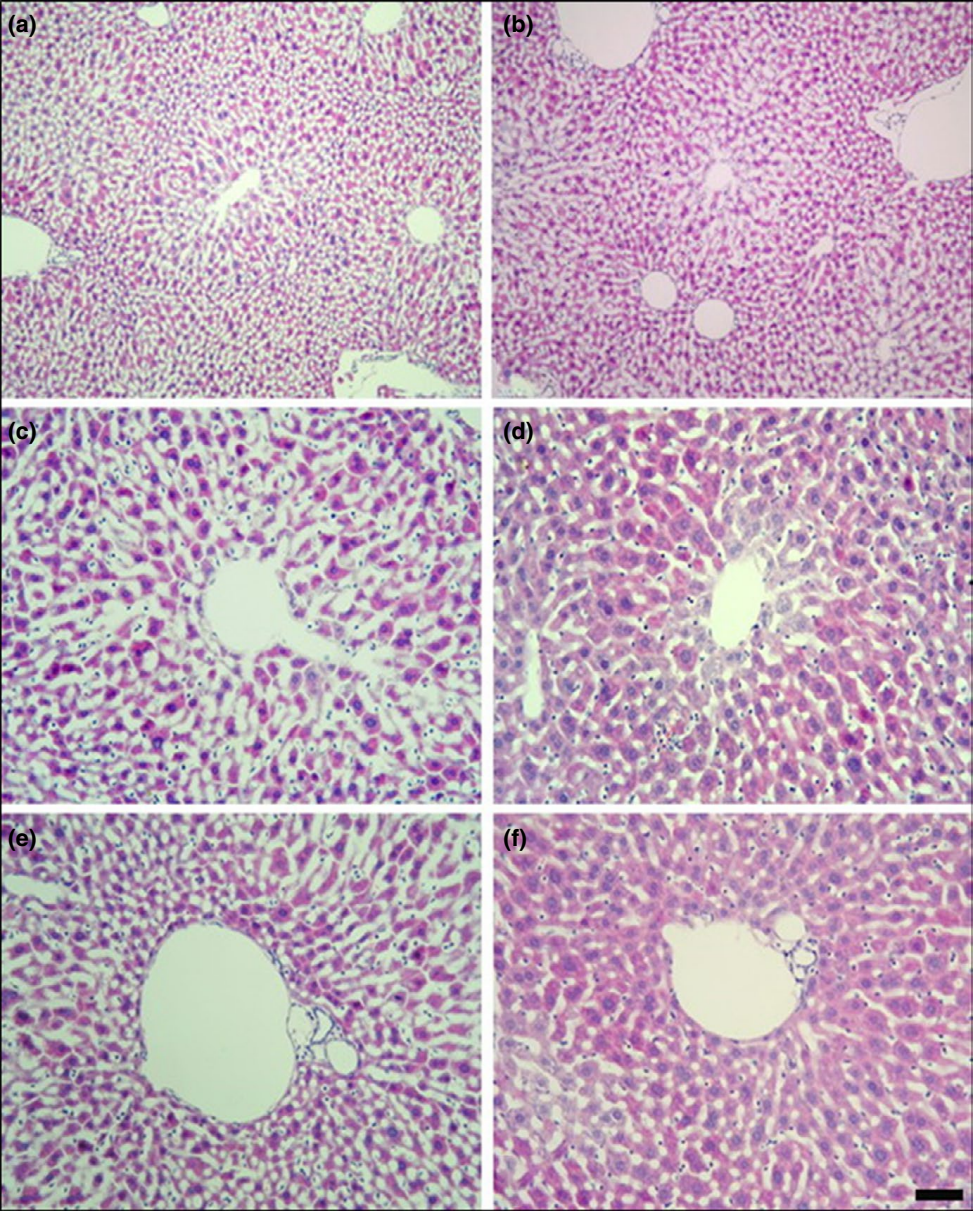
Photomicrography of livers of the mice of the control group (a, c, e) and treated with 1,000 mg/kg groups (b, d, f). Higher magnifications of central vein (c, d) and portal canal (e, f) of liver. Magnification: a, b: ×100, c–f: ×200. Scale bars: a, b: 100 μm, c–f: 50 μm

**Figure 5:**
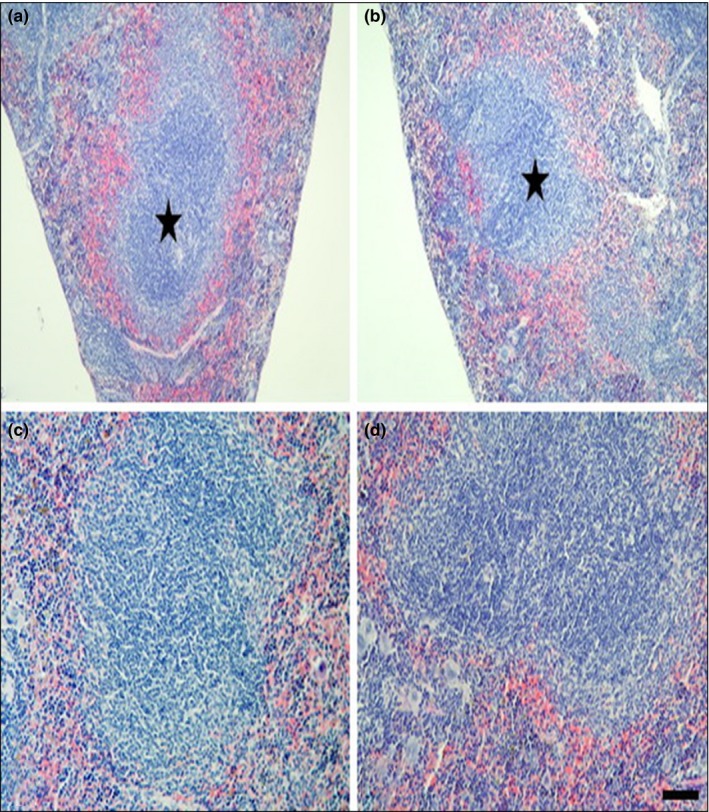
Photomicrography of spleens of the mice of the control group (a, c) and treated with 1,000 mg/kg groups (b, d). Higher magnifications of germinal center (c, d) of spleen. Magnification: a, b: ×100, c, d: ×200. Scale bars: a, b: 100 μm, c, d: 50 μm

Histopathological analysis of the liver, kidney, and spleen was performed in mice treated with high dose (1,000 mg/kg), and in mice from the control groups. The results showed normal morphological aspects, and the tissues maintained their histopathological architecture, as represented in Figures 3, 4 and 5.

## 4 DISCUSSION

Acute and subchronic oral toxicities of the nonpolar extract of licorice were investigated in this study through biochemical, hematological, and histological parameters. For centuries, herbal medicines and formulations have been considered to be safe, and this assumption has influenced their indiscriminate use in humans. The lack of proper dosage monitoring by experts and the lack of awareness of toxic effects also contributes to the indiscriminate use. Because of this, scientific knowledge toward oral toxicity for herbal medication is needed (Porwal et al., 2017).

Licorice is an herbal plant and has a history of being consumed for almost 6,000 years (Vaya, Belinky, & Aviram, 1997). Most of beneficial effects of licorice come from the hydrophobic compounds as well as the flavonoids and triterpenoids (Sun, Xie, Tian, & Liu, 2007). Nakagawa et al. (2010) reported that licorice polyphenols containing oil have a significant inhibitory effect on liver carcinogenesis.

Toxicity studies on herbal extracts are used to evaluate the possible risk of the chemical compounds in the plant that could result in adverse effects (Afolayan, Wintola, & Fouche, 2016). Multi‐dose mouse studies are often carried out during the developmental or safety testing phase of drug/chemical development, and during this time, an appropriate dose range is also studied. Acute toxicity is exposure to a chemical for <24 hr, whereas subchronic toxicity is the repeated administration of a chemical for 30 days or more (Hsu, Tsai, Chen, Huang, & Yen, 2011). The results of this study could provide information about the safety of licorice extract as well as the range of safe doses of licorice extract for the subsequent studies. In the present study, mice were orally administered extract of licorice at a dose of 2,000 mg/kg for acute toxicity. For subchronic toxicity experiments, the period of exposure was 120 days at doses of 50, 100, 500, 1,000 mg/kg.

Since glycyrrhizin was reported to induce hypertension, we measured the blood pressure in control and NERG‐treated groups before proceeding with the toxicity study. Although Isbrucker and Burdock (Isbrucker & Burdock, 2006) demonstrated the possibility of glycyrrhizin‐induced hypertension in rats, we saw no significant effect of NERG (500, 1,000 mg/kg) on blood pressure relative to the control group in mice (Table 1). From these results, we concluded that our NERG concentration (500, 1,000 mg/kg) for toxicity experiments would not cause hypertension in mice. We further concluded that the nonpolar extraction of licorice, which reduced the levels of glycyrrhizin in the extract, is responsible for the decreased effect of NERG on blood pressure in mice.

The acute toxicity study showed that the oral administration of NERG, up to 2,000 mg/kg of body weight, did not produce any signs of toxicity or death in mice (data not shown). These results suggest that the median lethal dose (LD50) of NERG is more than 2,000 mg/kg body weight via oral administration. The highest dosage suggested by the OECD 423 for animal welfare concern was 2,000 mg/kg of body weight. Therefore, our results demonstrate that this concentration of NERG (2,000 mg/kg body weight) has practically no toxicity via an oral route.

In the subchronic toxicity study, NERG showed neither signs of toxicity nor death. Measuring any reduction in body weight and internal organ weight is a simple and sensitive index of toxicity (Thanabhorn, Jaijoy, Thamaree, Ingkaninan, & Panthong, 2006). No significant differences in either of these measurements were observed at doses of 50, 100, 500, or 1,000 mg/kg of body weight during the 120 days of oral treatment (Figure 2). Likewise, organ weights were not significantly different in the lung, heart, kidney, spleen or testis (Table 2).

Aspartate aminotransferase and ALT, liver serum enzymes, are considered sensitive markers of hepatotoxicity and liver damage (Brondani et al., 2017). The levels of BUN and creatinine indicate renal dysfunction (Xu et al., 2013). We observed reduced levels of alkaline phosphatase and ALT activity in the serum of NERG‐treated mice compared to control mice (Table 3). There were no significant differences in the levels of AST, BUN, creatinine, direct bilirubin, glucose, magnesium, total bilirubin, triglycerides, or total protein. NERG treatment at 1,000 mg/ml showed significantly increased serum levels of albumin, calcium, cholesterol, HDL‐cholesterol, and iron relative to control mice. The hematological analysis is important because the hematopoietic system is one of the most sensitive targets of toxic compounds (Adewale et al., 2016). LY and MO showed significantly decreased values in NERG‐treated mice compared to the control group (Table 4). There were no significant changes, however, in WBC, NE, EO, BA, RBC, HB, HCt, MCV, MCH, and MCHC values between the control and NERG‐treated group (Table 4). Histopathological analysis did not show any abnormalities in the liver, spleen, or kidney, correlating with the results of the toxicity parameters tested. In this study, nonpolar licorice extract did not cause mortality or toxicity in mice. Furthermore, the licorice extract did not cause adverse effects on organ weight, serum biochemistry, hematological parameters, or histopathology.

## 5 CONCLUSIONS

The results of this study suggest that NERG, up to the dose of 1,000 mg/kg of body weight, does not show toxicity when administered orally in mice. This conclusion is deduced from the fact that acute and subchronic treatment with licorice extract did not cause any death or adverse effects in the mice and also did not induce significant alterations in any of the biochemical, hematological, or histopathological parameters investigated in this study. This is useful information for the medicinal use of *G. uralensis*. Further investigation on its therapeutic efficacy and biopharmaceutical application should be considered.

## CONFLICT OF INTEREST

The authors declare that there is no conflict of interest.

## ETHICAL APPROVAL

This study was approved by the Institutional Review Board of Hallym University.
